# Usefulness of C-Reactive Protein as a Marker for Prediction of Future Coronary Events in the Asian Indian Population: Indian Atherosclerosis Research Study

**DOI:** 10.1155/2010/389235

**Published:** 2010-06-28

**Authors:** Veena S. Rao, Natesha B. Kadarinarasimhiah, Shibu John, Sridhara Hebbagodi, Jayashree Shanker, Vijay V. Kakkar

**Affiliations:** ^1^Tata Proteomics and Coagulation Unit, Thrombosis Research Institute, India, Narayana Hrudayalaya Hospital, 560099 Bangalore, India; ^2^Elizabeth and Emmanuel Kaye Bioinformatics and Statistical Unit, Thrombosis Research Institute, 560099 Bangalore, India; ^3^Garry and Mary Weston Functional Genomics Unit, Thrombosis Research Institute, 560099 Bangalore, India; ^4^Thrombosis Research Institute, Manresa Road, Chelsea, SW36LR London, UK

## Abstract

Inflammation plays a pivotal role in all stages of atherosclerosis. Numerous inflammatory, lipid, and cytokines markers have been associated with coronary artery disease (CAD) risk but data directly comparing their predictive value are limited. Studies were carried to elucidate the role of high-sensitivity C-reactive protein (hsCRP), other inflammatory as well as lipid markers and their associations. Among 1021 subjects, comprising 774 CAD affected members from Indian Atherosclerosis Research Study (IARS), plasma hsCRP levels showed strong correlation with inflammatory markers, namely, IL6 (*r* = .373; *P* = <.0001), sPLA2 (*r* = .544; *P* = <.0001) as also with fibrinogen (*r* = .579; *P* = <.0001). Levels of hsCRP were higher among subjects affected by CAD who suffered a repeat coronary event as compared to those who remained event free and subjects in the top quartile of hsCRP (>3.58 mg/L) were found to have a fourfold higher risk. In conclusion, hsCRP appears to be an independent predictor of recurrent CAD events in Asian Indian population.

## 1. Introduction

Inflammation plays a major role in the pathophysiology of atherosclerosis [1–4]. Many novel inflammatory markers have been investigated in an effort to improve the risk predictive ability of future coronary artery disease (CAD) [5–8]. With the exception of high-sensitivity C-reactive protein (hsCRP), no inflammatory marker evaluated thus far has demonstrated consistent and independent value towards prediction of future CAD risk. High sensitive CRP has been advocated as the most attractive candidate novel bio-marker for inclusion in existing risk prediction models, primarily due to its stability, low diurnal variation, and ease of measurement [9]. hsCRP has been accepted to provide additional value to the Framingham risk scoring system devised for future 10yr CAD risk prediction, even in the presence of conventional CAD risk factors [10–13]. 

CRP is an acute-phase reactant belonging to the pentraxin family. It is almost exclusively produced in the hepatocytes under the control of cytokines [14], although extrahepatic sites of CRP synthesis have been reported [15–18]. The major functions of CRP include binding to various ligands on damaged tissue followed by propagation of both anti- and proinflammatory effects [19–21]. Recent evidence indicates that CRP plays an active role in atherosclerosis [22, 23]. There is general consensus that individuals in the top quartile of CRP have a two- to three-fold higher risk of an incident as well as future coronary event compared to those in the bottom quartile [24]. However, there is ongoing debate on the potential of plasma CRP to predict future coronary risk over and above that of established CAD risk factors. The existing reports are contradictory [6, 12, 24–35]. Significant race-based differences in CRP levels have been reported. Considering the prevalent lacunae in current knowledge, more so with respect to the Asian Indian population, this study was designed to assess the value of hsCRP in predicting future CAD in a cohort of high-risk Indians residing in the Indian subcontinent.

## 2. Methods

The Indian Atherosclerosis Research Study (IARS) is an ongoing, family-based, epidemiological study initiated in 2004 to investigate the prevalent genetic, traditional, and environmental factors associated with CAD in a cohort of Asian Indians in their home country. Novel biomarker discovery is one of the many specific objectives of this large-scale prospective study. The families in the IARS were enrolled from two cities: Bangalore in south India and Mumbai in western India. Subjects were recruited through a proband treated for CAD and its complications. A proband was eligible for recruitment when he was older than 60 (men) or 65 (women) years at onset of CAD. This was done in order to identify families with early onset CAD. All probands had a positive family history of CAD/CVD. We defined CAD-affected individuals as those who had documented evidence of an acute coronary syndrome with or without angiographic evidence of CAD and/or had undergone revascularization procedures. Probands' family members (both affected and unaffected by CVD) were subsequently enrolled into the study, provided they were aged 18 years or above at the time of recruitment. There was no upper age limit for any of the nonproband recruits. Subjects with present or past major illnesses such as cancer, cardiomyopathy, rheumatic heart disease, liver or renal disease and concomitant infection were excluded from the study. All participants gave their written informed consent to participate in the study, which was approved by the local ethics committee. 

A detailed case record form containing information on demographics, anthropometry, and medical history of diabetes, hypertension, and CVD was completed for all participants. A general physical examination was performed, along with measurement of vital parameters. Relevant information was obtained by personal interviews with subjects and from medical records. Prevalence of diabetes, hypertension, and CVD was ascertained based on self-report of physician's diagnosis and/or use of prescription medications along with medical records of diagnosis and therapy. Patients were followed up via telephone calls once every year. This follow-up data was collected during conversation with the patients themselves and all clinical data, including current medical therapy was judiciously recorded.

### 2.1. Biomarker Assays

All the biomarker assays were performed in accordance to standard protocols at the central research facility of the Thrombosis Research Institute (TRI) at Bangalore. Serum total cholesterol and triglyceride were estimated by standard enzymatic analysis using reagents, standards and controls from Randox Laboratories Ltd. (Antrim, UK). The concentrations of high-density lipoprotein (HDL)-cholesterol were estimated after precipitation of non-HDL fractions with a mixture of 2.4 mmol/L phosphotungstic acid and 39 mmol/L magnesium chloride. The concentrations of low-density lipoprotein (LDL)-cholesterol were calculated using the Fridewald formula. Apolipoproteins A1 and B100 (Orion Diagnostics, Espoo, Finland) were estimated by immunoturbidimetry method. Analyses were carried out on a Cobas-Fara II Clinical Chemistry Autoanalyser (F. Hoffmann La Roche Ltd., Basel, Switzerland). Three commercial controls purchased from Randox Laboratories, one from Orion Diagnostica for apolipoproteins and a normal human serum pool (NHP) prepared in-house was run with every batch of assays. The interassay coefficients of variation (CV) for the commercial controls and NHP ranged from 4.9% to 7.0% for total cholesterol, 6.1% to 7.7% for triglyceride, 7.1% to 12.2% for HDL-cholesterol, 3.3% to 5.2% for Lp(a), 9.9% to 14.2% for apolipoprotein A1, and 10.7% to 13.9% for apolipoprotein B100.

Plasma interleukin (IL)-6 level was measured by enzyme-linked immunosorbent assay (R & D Systems, Minneapolis, USA); the interassay CV for NHP was 4.3%. Plasma hsCRP level was measured using the Roche latex Tina quant kit (Roche Diagnsotics, Basel, Switzerland); the CV of NHP was 7.85%. Levels of secretory phospholipase A2 (sPLA2) were determined using a sandwich immunometric assay (Cayman Corporation, Michigan, USA) and the interassay CV of NHP was 5.37%. Plasma fibrinogen and FVII:C activity were measured in clotting assays on an Automated Coagulation Analyser (ACL 300, Instrumentation Laboratory, Milano, Italy). The reagents, calibrators, and controls were purchased from IL (Lexington, MA, USA). A commercial control and pooled normal human plasma (NHP) prepared in-house from citrate plasma of 30 healthy volunteers were run with every batch of assay. The interassay CV of the commercial control and NHP were 5.7% and 5.9%, respectively, for fibrinogen and 5.9% and 11.6%, respectively, for FVII:C. The values of fibrinogen and FVII:C for the commercial control were within the pre-assigned ranges in every batch of assay.

### 2.2. Statistical Analysis

All statistical analyses were performed using SPSS version 12. A *P*-value of less than.05 was considered statistically significant. Variables that showed a skewed distribution were log transformed before carrying out further statistical analysis. For the purpose of clarity, the untransformed mean and standard error of the mean (SEM) are shown in tables. Differences between continuous variables were assessed using Student's *t*-test and those between categorical variables using the chi square test. Analysis of covariance was also used to test for significance after adjusting for potential confounding variables. Odds ratios (ORs) and corresponding 95% confidence intervals (CIs) were calculated using conditional logistic regression analysis.

## 3. Results

### 3.1. Baseline Characteristics of Study Population

A total of 518 families comprising 2318 individuals were recruited into Phase I of the IARS.  Table 1 describes the baseline clinical and demographic characteristics of the study population in two groups: CAD-affected individuals who have suffered a coronary event and CAD unaffected people at the time of recruitment. Traditional coronary risk factors, namely, diabetes, hypertension and lipid profile were more prevalent in the affected group. The lower levels of total and LDL-cholesterol noted in the CAD-affected group may be due to the use of statins in over 70% of affected subjects.

### 3.2. Comparison of Baseline hsCRP Levels Among CAD-Affected and Unaffected Subjects

Fasting venous blood samples for biomarker analysis were collected from each one of the 2318 individuals recruited into phase 1 of the IARS. hsCRP levels were assayed in 1021 out of the above 2318 collected samples. The selection of samples for the study was random. Levels of CRP are known to vary across body mass index (BMI) and gender. However, adjustment for both of these variables as also for statins did not reveal a significant difference in the CRP titres between affected and unaffected individuals (Table 2). 

**Figure 1:**
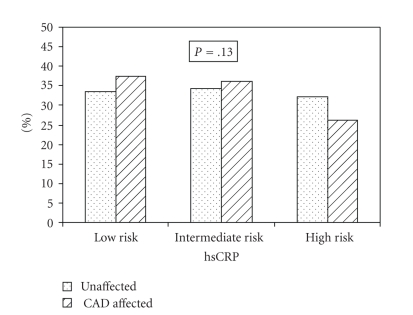
Classification of subjects based on high-sensitivity C-reactive protein (hsCRP) levels according to Joint American Heart Association and Centers for Disease Control and Prevention guidelines: low risk <1 mg/L, moderate risk 1–3 mg/L, and high risk >3 mg/L. CAD = coronary artery disease.

Figure 1 categorizes our study population according to the 2003 Joint American Heart Association and Center for Disease Control and Prevention (AHA-CDC) (Ref) guidelines for risk stratification of CAD based on CRP titres as follows: low risk <1 mg/L, moderate risk 1–3 mg/L, and high risk >3 mg/L. We found no significant difference in the distribution of subjects in the three risk classes between the CAD-affected and -unaffected subjects (*P* = .13), indicating that application of AHA-CDC guidelines for CRP classification may not hold true for all populations.

### 3.3. Correlation of hsCRP Levels with Inflammatory and Lipid Markers

The unadjusted and adjusted correlation coefficients for hsCRP are shown in Table 3. hsCRP significantly correlated with IL-6, fibrinogen and sPLA2 levels, after adjustment for age, gender, and BMI. There was moderate correlation with lipoproteins.

### 3.4. hsCRP and Risk of Future CAD Events

**Figure 2:**
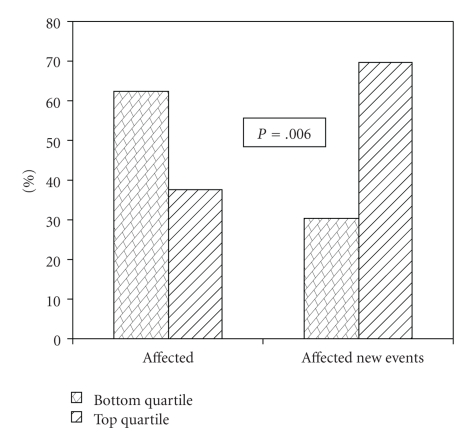
Distribution of subjects according to high-sensitivity C-reactive protein (hsCRP) quartiles.

The study cohort was periodically followed up over telephone at 1 year intervals. 28.3% of the original cohort was lost during the follow-up process. Detailed medical information was obtained in the remaining cases (71.7%). 115 individuals had a new coronary event, irrespective of their baseline disease status (CAD affected or unaffected). CAD-affected subjects who suffered a recurrent event (*n* = 91) had significantly higher levels of hsCRP than affected subjects who remained event free (3.98 ± 0.56 versus 2.9 ± 0.17 (mg/L); *P* = .009). Over 70% of CAD-affected individuals who suffered a repeat event were in the top quartile of hsCRP titres (Figure 2). 

Logistic regression model was used to determine the ability of hsCRP level to predict future coronary events based on quartile distribution. There was increasingly significant association of CAD risk with increasing quartiles of hsCRP levels. The odds of a recurrent coronary event for CAD-affected individuals in the top quartile of hsCRP was about four times that of individuals in the other three quartiles (95% CI 1.45–9.89; *P* = .006; Model 1, Table 4). After adjusting for traditional risk factors such as hypertension, diabetes, gender, age, and BMI, the significant association between hsCRP and risk of repeat coronary events remained for individuals in the top hsCRP quartile (OR 3.9, 95%CI 1.41–10.8; *P* = .009; Model 2, Table 4). After additional adjustment for IL-6, although the OR increased to over fourfold, the significance of the association with future CAD events was nominal (OR 4.359, 95% CI 1.023–18.573; *P* = .047; Model 3, Table 4). When IL-6 was excluded and lipid variables (total cholesterol, triglycerides, HDL and LDL) were added into the model, the association between the top quartile and risk of repeat CAD event persisted (OR 3.106 95% CI 1.07–9.014; *P* = .037; Model 4 and 5, Table 4.

## 4. Discussion

The increasing pressure on health resources worldwide, has led to the emphasis on preventive measures in the battle against the global pandemic of CAD [36, 37]. Indians are arguably at a higher risk of developing CAD than other races and this cannot be explained by traditional risk factors alone [38–42]. Although a multitude of factors have been measured in the blood of subjects with established atherosclerosis, only hsCRP has demonstrated an additive value to the risk assessment of CAD, as endorsed by the Framingham risk score [9, 11, 26].

The IARS is an ongoing large-scale family-based study that is investigating novel biomarkers that may contribute to enhanced risk of CAD in Asian Indians, thereby enabling the identification of high-risk subjects who may benefit from early intervention. Most published reports of hscRP levels in Asian Indians have been carried out on subjects living outside India [43–46]. There are relatively fewer studies in the Indian population [47, 48] that have reported relatively small cohorts. Thus, this is the first study to determine the utility of hsCRP as a marker for risk prediction in Asian Indian subjects with CAD living in their home country. 

We did not find a significant difference in baseline levels of hsCRP between CAD affected and unaffected subjects even after adjusting for gender, BMI, and statin therapy. This may possibly be attributed to therapeutic modifications in affected subjects in terms of treatment with inhibitors of arachidonic acid metabolism (aspirin), PPR*γ* antagonists amongst others that are known to possess anti-inflammatory properties in addition to their primary effect. Moreover lifestyle and diet modifications play a significant role in overall risk reduction. In our cohort, over 69% of the CAD affected subjects were on statin therapy while only 2.9% were on this drug in the unaffected group. As expected, hsCRP levels displayed significant correlation with inflammatory markers IL-6, sPLA2, fibrinogen, and neopterin (Table 3). Interestingly, when we compared the hsCRP levels in CAD-affected subjects who were followed up for 4 years over telephone to assess their cardiovascular health, we found that about 70% of those who developed a recurrent event were in the top hsCRP quartile (>3.58 mg/L). These individuals have approximately four times higher risk of developing a recurrent coronary event than those in the bottom quartile (OR 3.79, 95% CI 1.45–9.89; *P* = .006). This risk persisted even after adjusting for traditional risk factors such as gender, diabetes, hypertension, BMI, and lipids (Table 4, Model 5), indicating that hsCRP may be an independent predictor of recurrent CAD event in our population. This observation agrees with the published studies carried out in other ethnic groups. Over the last few years, multiple studies have shown an enhanced risk of recurrent coronary events with high hsCRP levels in normal healthy populations and also people within the various spectra of ischemic heart disease from sub clinical disease through stable angina to acute coronary syndromes in the presence and absence of other cardiovascular risk factors [6, 10, 12, 26–28, 31–35].

Our findings indicate that in the Asian Indian population, individuals in the top quartile of hsCRP have an approximately threefold higher risk of reinfarction, even after adjustment for traditional CAD risk factors thus implying that hsCRP may have additional utility for effective risk stratification in this high risk ethnic population. This may strengthen the case towards a recommendation of routine measurement of hsCRP along with lipids for improving risk prediction and identification of high-risk individuals. The AHA-CDC has devised guidelines for the classification of subjects as mild, intermediate and, high risk based on hsCRP levels. However, this classification is based on data obtained in Caucasian populations, and there is significant variation in hsCRP level based on ethnic origin [43, 44, 49–52]. High-sensitivity CRP levels are highest among blacks, followed by South Asians, Mexicans, Hispanics, and Caucasians. In addition, Asian Indians have a higher predisposition to diabetes and CVD, resulting in an inherent proinflammatory state [46]. Taking all of these facts into account, the feasibility of application of the AHA-CDC guidelines for classification of subjects needs to be tested before implementation in any population. Upon classifying our study population according to the AHA-CDC guidelines, we found no significant difference in the distribution of CAD affected and unaffected subjects across the three AHA-CDC groups (Figure 1). The unaffected subjects were distributed equally among the three groups. This supports the fact that population-specific reference intervals are required for accurate risk stratification. 

## 5. Conclusion

Our data indicate that high levels of hsCRP provide additional predictive value to the traditional risk factors in identififying CAD-affected subjects who could develop a recurrent coronary event. Hence, it appears that routine measurement of hsCRP levels may provide better stratification of CAD risk in this population. However, it is important to carry out large-scale population-based studies in order to modify and adapt the existing AHA-CDC guidelines so as to accurately specify CAD risk in various ethnic populations.

##  Author Contribution

V. S. Rao: concept, planning, study and assay design, data analysis and interpretation, study management and manuscript composition; N. B. Kadarinarasimhiah: biomarker assays; S. John and S. Hebbagodi: data analysis, J. Shanker: study management and manuscript revision V. V. Kakkar: concept, manuscript revision and overall guidance.

## Figures and Tables

**Table 1:** Baseline characteristics of study participants.

	CAD-patients (*n* = 774)	No CAD (*n* = 1544)	*P*
Continuous variables, mean (SD)			

Age	55.93 (9.36)	40.37 (14.15)	<.0001
BMI	25.83 (3.85)	25.67 (4.9)	.418
Systolic blood pressure (mmHg)	127.57 (16.74)	122.18 (15.35)	<.0001
Diastolic blood pressure (mmHg)	81.53 (8.55)	81 (9.26)	.186
Total cholesterol (mg/dL)*	155.41 (40.79)	178.27 (40.33)	<.0001
Triglycerides (mg/dL)*	149.84 (68.86)	131.98 (85.09)	<.0001
HDL-cholesterol (mg/dL)*	38.75 (9.06)	42.46 (10.6)	<.0001
LDL-cholesterol (mg/dL)*	86.27 (33.76)	110.18 (34.82)	<.0001

Categorical variables, *n* (%)			

Statin	529 (69.2)	44 (2.9)	<.0001
Male	630 (81.6)	724 (46.9)	<.0001
Female	142 (18.4)	820 (53.1)	<.0001
Smoking	⋯	⋯	⋯
Never	465 (60.5)	1356 (88.1)	⋯
Ex-smoker	241 (31.4)	61 (3.96)	⋯
Occasional	5 (0.7)	25 (1.62)	⋯
Current smoker	57 (7.4)	98 (6.36)	⋯

History of			

Diabetes	349 (67.9)	529 (57.8)	<.0001
Hypertension	378 (73.8)	659 (72.0)	.37

Probability values for continuous variables are from 2-sample *t*-tests and for categorical variables from chi square tests.

*Log-transformed distribution.

**Table 2:** Baseline high-sensitivity C-reactive protein (hsCRP) levels (mg/L).

Male			Female		
Unaffected (*n* = 281)	Affected (*n* = 321)	*P*	Unaffected (*n* = 336)	affected (*n* = 63)	*P*
2.596 ± 0.26	2.642 ± 0.239	.371*	3.303 ± 0.228	4.658 ± 0.664	.232*

hsCRP test performed in 1021 samples.

*adjusted for body mass index and statin use.

**Table 3:** Correlation of high-sensitivity C-reactive protein (hsCRP) levels with inflammatory markers and lipids.

	Unadjusted		Adjusted^†^	
Characteristics	Correlation	*P*	Correlation	*P*
sPLA2 (pg/mL)*	0.544	<.0001	0.464	<.0001
Interleukin 6 (pg/mL)*	0.373	<.0001	0.325	<.0001
Total cholesterol (mg/dL)	0.156	<.0001	0.11	<.0001
HDL (mg/dL)	−0.147	<.0001	−0.163	<.0001
LDL (mg/dL)	0.154	<.0001	0.114	<.0001
ApoA1 (g/L)	0.043	.168	0.029	.354
ApoB (g/L)	0.201	<.0001	0.17	<.0001
Triglyceride (mg/dL)*	0.208	<.0001	0.161	<.0001
Fibrinogen (g/L)	0.579	<.0001	0.522	<.0001
FVII:C (%)	0.215	<.0001	0.105	.001

*log-transformed distribution.

^†^Partial correlations adjusted for age, gender, body mass index, and smoking status.

sPLA2: secretory phospholipase A2; HDL: high-density lipoprotein; LDL: low-density lipoprotein; ApoA1: apolipoprotein A1; ApoB: apoplipoprotein B100; FVII:C: factor VII coagulant activity.

**Table 4:** Risk of recurrence of coronary artery disease (CAD) events based on high-sensitivity C-reactive protein (hsCRP) quartiles.

		hsCRP quartile				
		1 (<0.7 mg/L)	2 (0.71–1.7 mg/L)	3 (1.71–3.57 mg/L)	4 (>3.58 mg/L)	*P*
Model 1	OR (95% CI)	1	1.219 (0.406–3.656)	2.541 (0.954–6.773)	3.793 (1.454–9.898)	.019
	*P*		.724	.062	.006	
Model 2	OR (95% CI)	1	1.194 (0.395–3.614)	2.585 (0.947–7.054)	3.903 (1.411–10.8)	.029
	*P*		.753	.064	.009	
Model 3	OR (95% CI)	1	1.790 (0.406–7.882)	3.363 (0.806–14.04)	4.359 (1.023–18.573)	.17
	*P*		.441	.096	.047	
Model 4	OR (95% CI)	1	1.071 (0.346–3.316)	1.958 (0.695–5.517)	3.204 (1.113–9.227)	.093
	*P*		.905	.204	.031	
Model 5	OR (95% CI)	1	1.115 (0.363–3.429)	1.814 (0.636–5.172)	3.106 (1.07–9.014)	.131
	*P*		0849	.265	.037	

Model 1: Unadjusted.

Model 2: Adjusted for hypertension, diabetes, gender, age, and body mass index.

Model 3: Additional adjustment for interlukin-6*

Model 4: Additional adjustment total cholesterol, high-density lipoprotein (HDL) and triglyceride*.

Model 5: Additional adjustment total cholesterol, HDL, triglyceride* and low-density lipoprotein.

*log-transformed distribution.

OR: odds ratio; CI: confidence interval.
